# Endoscopic submucosal dissection for early gastric cancer, using a disposable endoscope

**DOI:** 10.1055/a-2109-0778

**Published:** 2023-07-13

**Authors:** Kenichiro Okimoto, Tomoaki Matsumura, Mai Fujie, Naoki Akizue, Keisuke Matsusaka, Jun Kato, Naoya Kato

**Affiliations:** 1 Department of Gastroenterology, Graduate School of Medicine, Chiba University, Japan; 2 Endoscopy Center, Chiba University Hospital, Japan; 3 Department of Diagnostic Pathology, Graduate School of Medicine, Chiba University, Chiba, Japan

The environment in reusable endoscopes is conducive to bacterial growth 1 2 because of difficult-to-clean areas, deterioration with reprocessing, and surface abrasion. Hence, the risk of cross-infection has been reported 3 . Single-use endoscopes can solve these problems. Diagnostic esophagogastroduodenoscopy using a novel sterile single-use disposable endoscope (Ambu aScope Gastro; Ambu, Ballerup, Denmark) has been reported in recent years ( Fig. 1 ) 4 .

**Fig. 1:**
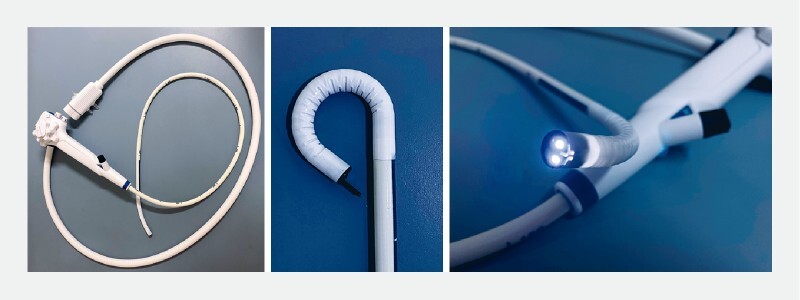
The Ambu aScope Gastro single-use device: diameter 9.9 mm; working channel 2.8 mm; bending angle, up 210°, down 90°, left 100°, right 100°; and equipped with two light-emitting diodes (LEDs).

Endoscopic submucosal dissection (ESD) is widely performed for early-stage gastric cancer regardless of tumor size, morphology, and location. ESD is minimally invasive and can achieve high en bloc and complete resection rates 5 . Here, we present a case of early gastric cancer resected by means of ESD using the abovementioned single-use disposable endoscope ( Video 1 ).

**Video 1** Endoscopic submucosal dissection (ESD) of an early gastric cancer by means of a single-use disposable endoscope.


A 70-year-old woman with early gastric cancer was referred for ESD. The tumor (5 mm, 0-IIc) was located at the anterior wall of the greater curvature of the middle body. The tumor was well-demarcated by magnified narrow band imaging (NBI) ( Fig. 2 ). A DualKnife J 2.0 mm (Olympus Medical Systems, Tokyo, Japan) with magnified NBI via a GIF-XZ1200 (Olympus) was used for marking. The endoscope was changed for the single-use disposable endoscope when marking had been done, and the ESD procedure was performed using the single-use scope. Imaging and maneuverability were adequate for performing mucosal incision and submucosal dissection ( Fig. 3 a ). The clip-and-line traction method was successfully applied by means of the 2.8-mm working channel ( Fig. 3 b ). In addition, the bleeding point could be identified using the waterjet function of the device ( Video 1 ). Hence, the tumor was completely resected ( Fig. 3 c ) without any major complications. The pathological finding was adenocarcinoma of fundic gland type, SM1, Ly0, V0, HM0, and VM0, with curative resection.

**Fig. 2:**
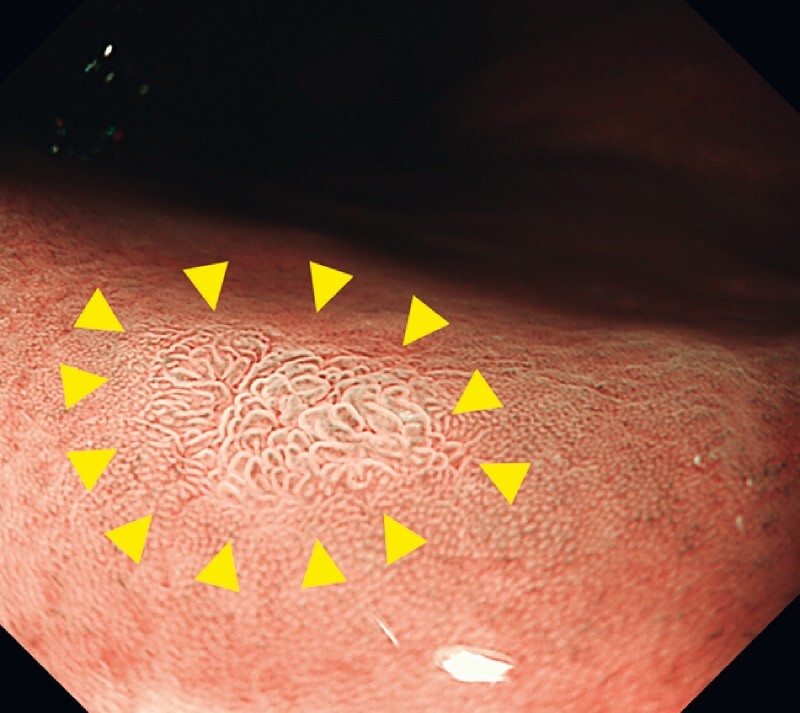
Magnified narrow-band imaging (NBI) image of early gastric cancer (yellow arrowheads), using a GIF-XZ1200.

**Fig. 3:**
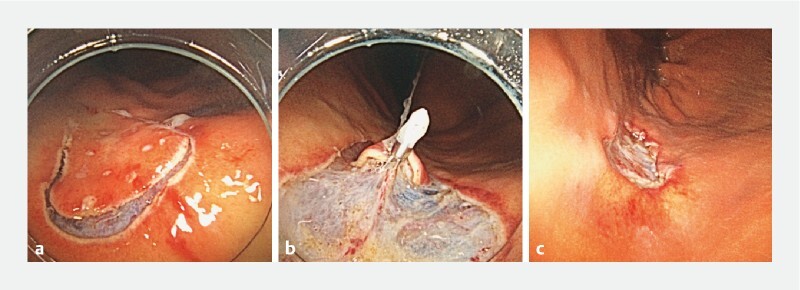
After marking had been done, gastric endoscopic submucosal dissection (ESD) was performed employing the single-use gastroscope. **a** Mucosal incision. **b** Clip-and-line traction was successfully applied. **c** The tumor was completely removed.

Our case demonstrates a successful gastric ESD with a single-use disposable scope. This device could be considered as an alternative to reusable endoscopes if an appropriate case is selected.

Endoscopy_UCTN_Code_TTT_1AO_2AC

## Notes

Endoscopy E-Videos

https://eref.thieme.de/e-videos
*E-Videos* is an open access online section of the journal *Endoscopy* , reporting on interesting cases and new techniques in gastroenterological endoscopy. All papers include a high-quality video and are published with a Creative Commons CC-BY license. Endoscopy E-Videos qualify for HINARI discounts and waivers and eligibility is automatically checked during the submission process. We grant 100% waivers to articles whose corresponding authors are based in Group A countries and 50% waivers to those who are based in Group B countries as classified by Research4Life (see: https://www.research4life.org/access/eligibility/ ). This section has its own submission website at https://mc.manuscriptcentral.com/e-videos
